# Immunoglobulin Y antibodies against colonization-related genes block the growth and infection of *Helicobacter pylori*

**DOI:** 10.3389/fimmu.2025.1582250

**Published:** 2025-06-18

**Authors:** Shiyuan Deng, Xiaoling Luo, Yunxiao Du, Rania G. Elbaiomy, Weihan He, Rong Guo, Ahmed H. El-Sappah, Xiaohong Jian, Yongmei Xie, Mohammed Bakeer, Zaixin Li, Zhi Zhang

**Affiliations:** ^1^ Department of Biological Engineering, Sichuan University of Science & Engineering, Zigong, China; ^2^ Department of Gastroenterology, Fushun People’s Hospital, Zigong, China; ^3^ Department of Genetics, Faculty of Agriculture, Zagazig University, Zagazig, Egypt; ^4^ State Key Laboratory of Biotherapy and Cancer Center, West China Hospital, Sichuan University, Chengdu, China; ^5^ Division of Hematology and Medical Oncology, Mayo Clinic, Jacksonville, FL, United States; ^6^ Division of Internal Medicine-Clinical Hematology, Al-Azhar University, Cairo, Egypt

**Keywords:** immunoglobulin Y, *Helicobacter pylori*, growth, infection, eradication rate

## Abstract

**Introduction:**

Immunoglobulin Y (IgY) has emerged as a promising antibody therapy for the eradication of *Helicobacter pylori* (*H. pylori*) independent of antibiotics. However, the roles and differences of IgY antibodies targeting various genes against *H. pylori* remain unclear.

**Methods:**

The recombinant antigens of five colonization-related genes — *FlaA*, *BabA2*, *NapA*, *HpaA*, and *UreB* — are prepared using a prokaryotic expression system and then subject to immunize laying hens for IgY production. Subsequently, their biological activities are evaluated, including blocking bacterial growth, attenuating infection in GES-1 cells, and eradicating *H. pylori* in gastritis mouse models.

**Results:**

These IgY antibodies can recognize the full-length antigens of *H. pylori* and exhibit a direct inhibitory effect on the growth and infection of *H. pylori* with dose-dependent characteristics. Among these, anti-*FlaA* IgY shows greater antibacterial activity in inhibiting *H. pylori* growth and preventing adhesion to GES-1 cells. Oral administration of these IgY antibodies for two weeks (20.0 mg·kg^−1^·day^−1^) achieves a 25% to 37.5% eradication rate of *H. pylori* infection in mice. Interestingly, combination treatment with these IgY antibodies, based on their different roles, enhances antibacterial benefits and significantly promotes the recovery of gastrointestinal function.

**Conclusion:**

Our results indicate that IgY antibodies against colonization-related genes can directly block the growth and infection of *H. pylori*, and combination treatment with these antibodies offers more advantages in combating *H. pylori*.

## 1 Introduction

*H. pylori* is the leading cause of chronic and atrophic gastritis, peptic ulcers, gastric lymphoma, and gastric carcinoma. Successful colonization is crucial for *H. pylori* infection, which involves various genes, including urease (1, 2), flagella (3), blood-group antigen-binding adhesin (*BabA*) (4), *H. pylori* adhesin A (*HpaA*) (5), and neutrophil-activating protein (*NapA*) (6). Each of these genes plays distinct roles during infection, such as facilitating bacterial movement, neutralizing gastric acid, and enhancing adhesion and infection in the stomach’s highly acidic environment. These genes are considered potential targets for developing various vaccines and treatments for *H. pylori* eradication. Among these, immunoglobulin Y (IgY) has emerged as a promising antibody therapy for *H. pylori* eradication.

IgY is an antibody produced by B lymphocytes after immunizing laying hens with specific antigens. It is enriched in egg yolk, yielding over 100 mg of IgY per egg. IgY is structurally and functionally similar to human IgG, and it has been widely used in the diagnosis and treatment of both animal and human diseases due to its high stability, specificity, safety, and efficacy (7–10). The application of IgY to the eradication therapy of *H. pylori* has been of great interest to researchers. Several laboratories developed IgY antibodies against *H. pylori* in the early stages based on whole bacteria or urease subunit B *(UreB)* antigens (11–13). Although it can significantly improve patients’ clinical symptoms, IgY does not exhibit the same efficacy as antibiotics in eradicating *H. pylori* infection, which limits its clinical development. In recent years, researchers have explored new targets for developing IgY antibodies and have verified their anti-*H. pylori* effect (1, 12, 14), thanks to a deeper understanding of the mechanisms of *H. pylori* infection.

As research on the mechanisms of *H. pylori* infection deepens, more genes will be identified as potential targets for IgY antibodies. However, their anti-bacterial roles and differences against *H. pylori* remain unclear, as does the question of whether it is more effective to eradicate *H. pylori* when these antibodies are combined at the same dose or lower doses. To address these questions, IgY antibodies targeting five different colonization-related genes—*FlaA*, *BabA2*, *NapA*, *HpaA*, and *UreB*—are prepared, and their antibacterial activities are systematically evaluated under the same experimental conditions, both *in vivo* and *in vitro*. Our research will provide new insights for developing combined treatment regimens using IgY antibodies to enhance efficacy in combating *H. pylori* infection.

## 2 Materials and methods

### 2.1 Materials

Protein Marker, DNA Marker, 2× PCR Mix Reagent, and Polyvinylidene Fluoride (PVDF) membranes were purchased from Thermo Fisher Scientific Inc. Selective medium for *H. pylori* culture (No. HB8647), trimethoprim (TMP) (No HB8646a), diaminobenzidine (DAB) substrate solution, and 3,3’,5,5’-tetramethylbenzidine (TMB) substrate solution, were obtained from Beijing Solarbio Science & Technology Co., Ltd. Isopropyl-β-D-thiogalactoside (IPTG) and Ni-NTA agarose purification resins were acquired from Shanghai BioEngine Sci-Tech Co., Ltd. Kanamycin and ampicillin were purchased from Biosharp. The HRP-conjugated goat anti-chicken IgY and HRP-conjugated rabbit anti-mouse IgG were both sourced from Sigma-Aldrich, while the FITC-conjugated goat anti-chicken IgY was obtained from AbBox solution Co., Ltd.

The recombinant DNA and its plasm ids, including pET-32a(+)-*BabA2*, pET-32a(+)-*UreB*, pET-32a(+)-*HpaA*, pET-28a(+)-*FlaA*, and pET-28a(+)-*NapA*, were synthesized and constructed by the General Bio (Anhui) Co., Ltd. The recombinant DNA sequences of *BabA2*, *UreB*, *HpaA*, *FlaA*, and *NapA* are provided in the supplementary files. The *H. pylori* strain (ATCC 700392) and the clinical isolate (generously gifted by Dr. Juan Liao at Sichuan University) are stored in our lab.

### 2.2 Animal

Female BALB/c mice (n=120) at 6 weeks of age were acquired from Chengdu Dashuo Laboratory Animal Co., Ltd (Sichuan Province, China). The animals were housed in controlled environmental conditions with room temperature (RT) regulated at 22 ± 3°C and humidity levels maintained at approximately 56% (± 10%). A standardized 12 h light/dark cycle (08:00-20:00 light phase) was implemented throughout the study period. *Ad libitum* access to water and standard laboratory feed was provided. Body mass measurements were systematically recorded at three-day intervals using calibrated analytical balances. Following a 7-day acclimatization period to ensure physiological stabilization, experimental protocols were initiated. All procedures involving animal subjects were conducted in accordance with the ethical guidelines approved by Sichuan University’s Institutional Animal Care and Use Committee, IACUC (Ethical Approval ID: 20230307036). For animal euthanasia, BALB/c mice were injected intraperitoneally with pentobarbital sodium, following the European Directive 2010/63/EU.

### 2.3 Preparation of recombinant antigens

Five recombinant plasmids, including pET-32a(+)-*BabA2*, pET-32a(+)-*UreB*, pET-32a(+)-*HpaA*, pET-28a(+)-*FlaA*, and pET-28a(+)-*NapA*, were individually transformed into BL21 (DE3) competent cells, respectively, and the positive transformants were verified by colony PCR. After identification, the expression and purification of recombinant antigens were conducted as previously described (15). The recombinant antigens were induced under 1.0 mM IPTG for 6 h. Then, the *BabA2*, *UreB*, *FlaA*, and *NapA* antigens were purified and renatured by washing and gradient dialysis with urea, while the *HpaA* antigen was purified using Ni^2+^-affinity chromatography. All prepared recombinant antigens were further analyzed by SDS-PAGE.

### 2.4 Immunization and IgY antibodies preparation

For immunization studies, 42-day-old Leghorn chickens (n = 36) were acclimatized in controlled isolators with 12 h photoperiod regulation, maintaining 23°C (± 2°C) RT and 75% (± 5%) humidity. Nutritional requirements were met with a standardized poultry diet and automated watering systems. For primary immunization, the five recombinant antigens (adjusted to 1.0 mg/mL) were emulsified with an equal volume of Complete Freund’s Adjuvant (CFA), with 200 μL administered per antigen. Subsequent booster immunizations utilized Incomplete Freund’s Adjuvant (IFA) following the same emulsification protocol. The thirty chickens were then randomly divided into five groups (six chickens per group) and immunized with *FlaA*, *UreB*, *BabA2*, *NapA*, and *HpaA*, respectively. The remaining 6 chickens are used for PBS immunization. Immunizations were performed intramuscularly at two sites on the breast at 21 weeks of age, followed by two booster immunizations at two-week intervals. Eggs were collected daily for two weeks after the final immunization. IgY antibodies were prepared from egg yolks using the water-dilution method, as previously described (16). Next, recombinant antigens *BabA2, UreB, FlaA, NapA*, and *HpaA* were individually coated (1.0 μg per well) and used to determine their corresponding antibody titers via ELISA, as previously described (17).

### 2.5 Effects of IgY antibodies on the growth of *H. pylori*

The preparation of *H. pylori* was done using an *H. pylori* selective medium. To investigate the effect of each IgY antibody on the growth of *H. pylori*, 50 μL of *H. pylori* solution (5 × 10^7^ CFU/mL) was added to each well of a 96-well plate. The concentrations of each IgY antibody were adjusted to 6 mg/mL, 4 mg/mL, and 2 mg/mL using the *H. pylori* selective medium. Then, 50 μL of each IgY antibody solution was added to the corresponding wells, mixed thoroughly, and co-incubated under anaerobic conditions at 37°C. After 24 h, the culture from each well was plated onto *H. pylori* selective solid medium and further incubated anaerobically at 37°C for 3–5 days. Colony counts were performed using the dilution plate count method, and the inhibitory rate was calculated. Furthermore, among these five antibodies (anti-*FlaA* IgY, anti-*BabA2* IgY, anti-*UreB* IgY, anti-*NapA* IgY, and anti-*HpaA* IgY), any three were selected and combined in equal concentrations (1:1:1 ratio) without changing the total antibody concentration, forming 10 combinations (**Table 1**). These combinations were then used to conduct combined antibacterial experiments.

**Table 1:** IC50 values of antibacterial activity of IgY combinations against *H. pylori in vitro*.

IgY Combination groups	IC50 values (mg/mL)
FUB	0.90
UBN	1.14
UBH	1.2
FBN	1.26
FBH	1.05
BHN	1.59
FUN	0.84
FUH	1.02
UHN	1.41
FHN	0.96

Among these IgY antibodies, including anti-*FlaA* IgY (F), anti-*UreB* IgY (U), anti-*BabA2* IgY (B), anti-*NapA* IgY (N), and anti-*HpaA* IgY (H), any three were selected and combined in equal concentrations (1:1:1 ratio), forming 10 combinations. For example, FUN was defined as a combination of anti-*FlaA* IgY, anti-*UreB* IgY, and anti-*NapA* IgY. These combinations were then used to conduct combined antibacterial experiments.

### 2.6 Immunofluorescence staining

GES-1 cells were seeded at a density of 3×10^4^ cells/well in 96-well plates and cultured overnight at 37°C in a 5% CO_2_ incubator. Each IgY antibody concentration was adjusted with DMEM medium and added to the 96-well plate for co-incubation with cells at a working concentration of 2 mg/mL. *H. pylori* were subsequently added to the wells at 1.0 × 10^6^ CFU/well and co-cultured with GES-1 cells for a 12 h infection period. Subsequently, immunofluorescence staining was conducted to observe the infection of GES-1 cells by *H. pylori*. Briefly, 200 μL of primary IgY antibody solution (a mixture of anti-*FlaA* IgY, anti-*UreB* IgY, anti-*BabA2* IgY, anti-*HpaA* IgY, and anti-*NapA* IgY in a ratio of 1:1:1:1:1, with a total concentration of 50 μg/mL) containing 3% FBS was added into wells and incubated at RT for 2 h. After three additional PBS washes, a secondary antibody of FITC-labeled goat anti-chicken IgY (diluted 1:250) was added and incubated at 4°C for 45 minutes in the dark. Finally, the wells were washed five times with PBS, 200 μL of DAPI (1.0 μg/mL) was added for 5 minutes at RT, the staining solution was discarded, and the samples were washed with PBS again. Finally, GES-1 cells were observed and photographed under a fluorescence microscope, and the ImageJ software was used for calculating and analyzing images.

### 2.7 Establishment of *H. pylori*-infected mice model

The *H. pylori* strain (ATCC 700392) was used to establish a mouse gastritis model. Mice (n = 110) underwent a 7-day gastric preconditioning protocol involving daily oral gavage with 0.1 mL of 3% sodium bicarbonate solution, followed 10 minutes later by administration of 0.1 mL of 50% ethanol solution. On day 8, mice were fasted for 12 h or overnight, then gavaged with 0.1 mL of 3% sodium bicarbonate solution, followed 10 minutes later by 0.2 mL of a bacterial solution containing 5 × 10^9^ CFU/mL of *H. pylori*. Food and water were resumed 2 h post-infection. *H. pylori* infection was performed by gavage every 3 days for 3 weeks. One week after the final infection (day 35), fecal samples were analyzed using rapid urease testing to confirm *H. pylori* infection. Five test-positive mice were randomly selected for euthanasia with intraperitoneal injection of pentobarbital sodium and dissection, and gastric mucosal fluid was inoculated onto solid culture media and incubated for 5 days. The infection status of these mice was further evaluated based on the *H. pylori* culture results.

### 2.8 Dietary IgY antibodies and eradication rate evaluation

*H. pylori*-infected mice (n = 72) were randomly divided into nine treatment groups of 8 mice each: PBS vehicle treatment group, amoxicillin treatment group, anti-*BabA2* IgY treatment group, anti-*FlaA* IgY treatment group, anti-*NapA* IgY treatment group, anti-*UreB* IgY treatment group, anti-*HpaA* IgY treatment group, a combination treatment group with FUN (including anti-*FlaA* IgY, anti-*UreB* IgY, and anti-*NapA* IgY), and a combination treatment group with FUB (including anti-*FlaA* IgY, anti-*UreB* IgY, and anti-*BabA2* IgY). On day 36, IgY antibodies treatments were administered by gavage at a dose of 20.0 mg/kg of IgY antibody or its combinations, given once daily for 2 weeks. On day 51, treatments were terminated, and fresh feces were collected from the mice. A rapid urease test (RUT) was conducted to evaluate the eradication rate of *H. pylori*. Additionally, a non-infected mouse group (n = 8) served as the normal control.

### 2.9 Evaluation of gastric emptying rate and intestinal propulsion rate

After collecting the feces, the mice were fasted for 12 h, after which a carbon ink solution was administered by gavage at a dose of 0.1 mL per mouse. Euthanasia was performed 30 minutes later, and the stomach, small intestine, cecum, and colon were collected. The total weight and net weight of the stomach were recorded first. The gastric emptying rate was evaluated based on the residual rate of gastric contents using the formula: gastric content residual rate (%) = [(gastric weight - gastric net weight)/carbon ink dosage] × 100%. Next, the distance from the pylorus to the carbon ink and the total length of the intestine were measured for each group of mice. The propulsion rate (%) was calculated as (distance of carbon ink propulsion in the small intestine (cm)/total length of the small intestine (cm)) × 100%.

### 2.10 Histopathological examination

The stomach was fixed in a 4% paraformaldehyde solution. Paraffin-embedded tissue slides were prepared with 3 µm and stained with hematoxylin-eosin (HE), and then tissue damage and inflammation were examined and photographed under a light microscope. To objectively evaluate inflammation, four non-overlapping microscopic fields from three independent tissue sections per mouse were randomly selected. These submucosal inflammatory cell infiltrations were determined independently by two pathologists. Data were normalized to the submucosal area (mm²) measured via ImageJ’s polygon tool, and results were expressed as mean ± SEM cells/mm².

### 2.11 Serum anti-*H. pylori* antibody detection by ELISA

After euthanasia, whole blood was collected and allowed to clot at RT for 3 h. The clotted sample was then centrifuged at 500 ×g for 5 minutes to pellet cellular debris, after which the supernatant serum was carefully aspirated using a pipette. The antisera against *H. pylori* were quantified using indirect ELISA. Briefly, the coating antigens comprised five purified recombinant antigens: *FlaA*, *UreB*, *BabA2*, *HpaA*, and *NapA*, totaling 1 μg per well. After blocking the plates with 5% skim milk in PBST for 2 h at RT, they were incubated with mouse serum diluted at 1:50 for 2 h at RT. Next, washed with PBST and then incubated with HRP-conjugated rabbit anti-mouse IgG for 1 h at 37°C. After an additional wash with PBST, TMB solution was added to each well. The plates were then incubated in the dark for 10 minutes, after which the reaction was terminated by adding H_2_SO_4_ to each well. The value was measured at 450 nm using a microtiter plate reader.

### 2.12 Statistical analysis

Data are expressed as the mean ± SEM derived from triplicate independent trials. Statistical comparisons were conducted through one-way ANOVA (SPSS 22.0) among treatment groups, with significance thresholds set at *p < 0.05 (significant) and **p < 0.01 (very significant).

## 3 Results

### 3.1 Preparation of recombinant antigens

**Figure 1:**
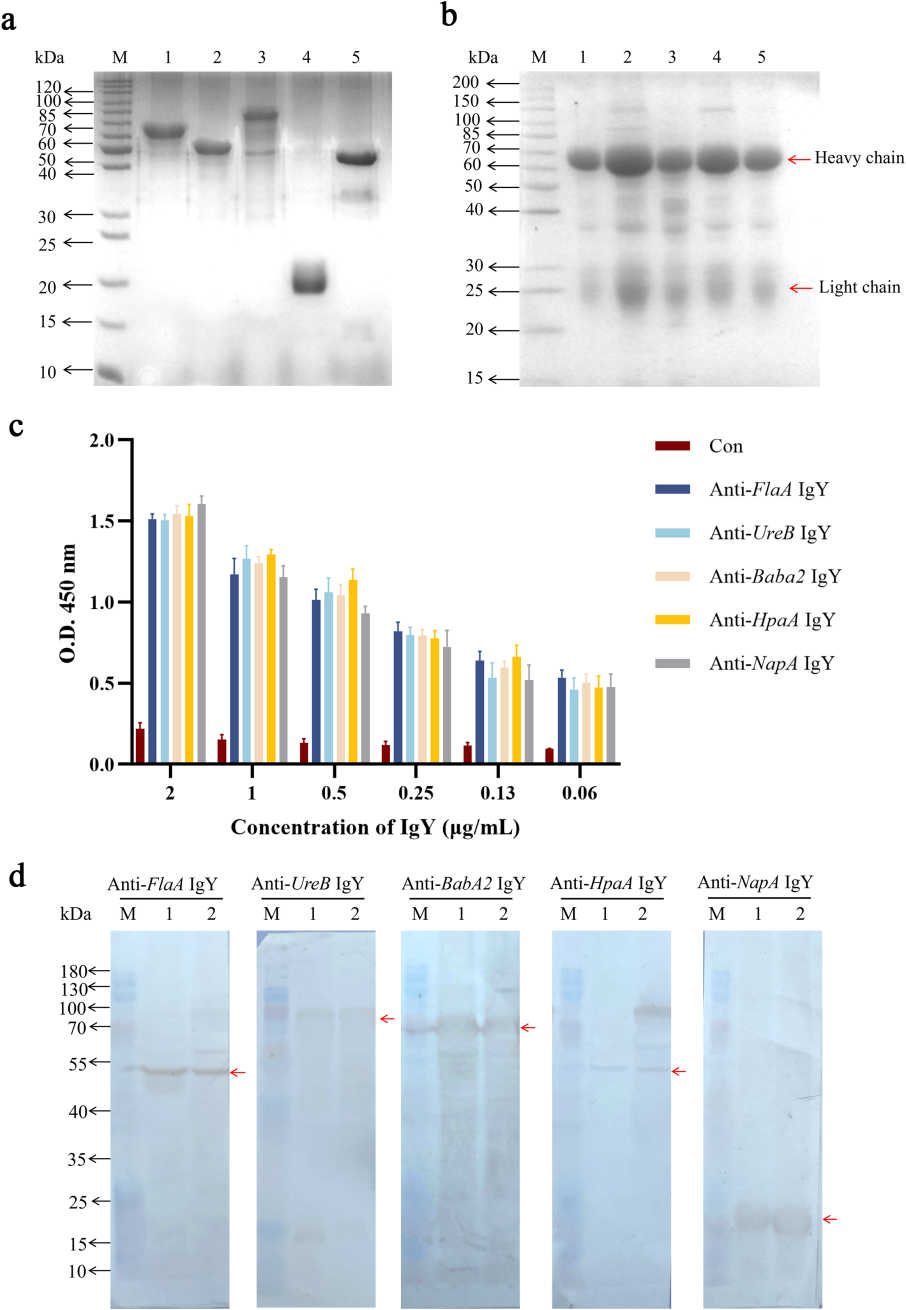
Preparation and identification of IgY antibodies. **(a)** SDS-PAGE analysis of the purified recombinant antigens. Lane M: the standard protein marker; Lanes 1 to 5: the purified recombinant antigens of *BabA2*, *FlaA*, *UreB*, *NapA*, and *HpaA* after purification, respectively. **(b)** SDS-PAGE analysis of the isolated IgY antibodies from egg yolk. Lane M: the standard protein marker; Lanes 1 to 5: the isolated IgY antibodies of anti-*BabA2* IgY, anti-*UreB* IgY, anti-*FlaA* IgY, anti-*NapA* IgY, and anti-*HpaA* IgY, respectively. **(c)** ELISA analysis of isolated IgY antibodies using purified recombinant antigens as coating antigens. The data are presented as means ± SEM (n = 4). Con, PBS-IgY Control. **(d)** The binding abilities of IgY antibodies, including anti-*FlaA* IgY, anti-*UreB* IgY, anti-*BabA2* IgY, anti-*HpaA* IgY, and anti-*NapA* IgY, to the whole bacterial antigens of two *H*. *pylori* strains, including *FlaA*, *UreB*, *BabA2*, *HpaA*, and *NapA*, were evaluated by using Western Blotting, respectively. Lane M: the standard protein marker; Lane 1: the standard *H*. *pylori* strain (ATCC 700392); Lane 2: clinical isolate of *H*. *pylori*.

After transformation with five individual plasmids containing recombinant DNA of *BabA2, FlaA, UreB, NapA*, and *HpaA*, the corresponding positive transformants were identified by colony PCR using specific primers (**Supplementary Figure 1A**). After 6 h of IPTG induction with 1.0 mM, the results of SDS-PAGE showed that five recombinant antigens were successfully expressed, and their molecular weights were consistent with the expected sizes (**Supplementary Figure 1B**). After purification, specific protein bands corresponding to *BabA2, FlaA, UreB, NapA, and HpaA* were observed at approximate sizes of 65 kDa, 55 kDa, 80 kDa, 20 kDa, and 45 kDa, respectively, as shown in **Figure 1a**.

### 3.2 Purification and identification of IgY antibodies

IgY antibodies were extracted using the water dilution method, and the results of SDS-PAGE showed that these IgY antibodies containing the respective anti-antigen (*BabA2*, *UreB*, *FlaA*, *NapA*, and *HpaA*) were successfully extracted. These IgY antibodies showed two major bands at approximately 35 KDa and 70 KDa, which corresponded to the light- and heavy-chain of IgY antibody, respectively (**Figure 1b**). Further ELISA analysis showed that the titers of these IgY antibodies reached up to 0.06 μg/mL (P/N ratio greater than 2.1) (**Figure 1c**). These antibodies can also recognize the corresponding recombinant antigens as well as the *H. pylori* whole bacterial antigens at the expected molecular weights determined by Western Blot (**Figure 1d**, **Supplementary Figure 2**).

### 3.3 Inhibitory effect of IgY antibodies on the growth of *H. pylori*

**Figure 2:**
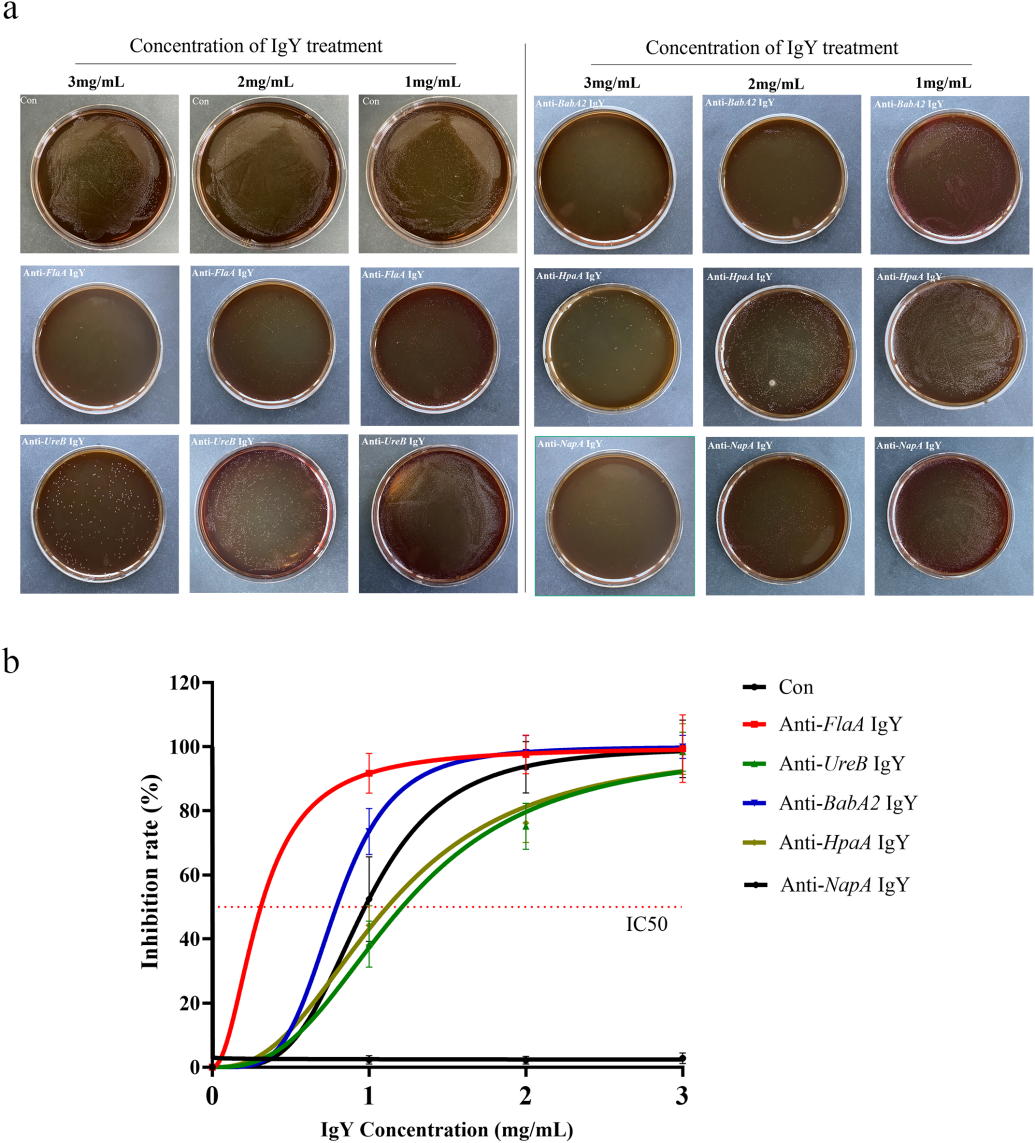
Inhibition rate of IgY antibodies on the growth of *H*. *pylori*. **(a)** Different concentrations of IgY antibodies (anti-*FlaA* IgY, anti-*UreB* IgY, anti-*BabA2* IgY, anti-*HpaA* IgY, and anti-*NapA* IgY) were co-cultured with *H*. *pylori* as described in the Materials and Methods section. **(b)**The dilution plate count method was used for colony counting, and the inhibitory rate was calculated. Data are presented as mean ± SEM (n=4). Con, PBS-IgY Control.

The antibodies, including anti-*FlaA* IgY, anti-*BabA2* IgY, anti-*UreB* IgY, anti-*NapA* IgY, and anti-*HpaA* IgY, were co-cultured with *H. pylori*, respectively. As shown in **Figure 2**, the growth of *H. pylori* could be significantly inhibited by IgY antibodies at different concentrations, exhibiting dose-dependent characteristics. Compared to other IgY antibodies, anti-*FlaA* IgY demonstrated the greatest inhibitory effect on *H. pylori*, while anti-*UreB* IgY exhibited the weakest antibacterial effect on *H. pylori*.

Furthermore, among these five IgY antibodies, any three were selected and combined in equal concentrations (l:1:1 ratio)to conduct a combined antibacterial experiment. As shown in **Table 1**, the FUN combination (including anti-*FlaA* IgY, anti-*UreB* IgY, and anti-*NapA* IgY) and the FUB combination (including anti-*FlaA* IgY, anti-*UreB* IgY, and anti-*BabA2* IgY) exhibited the strong antimicrobial advantage *in vitro*, with IC50 values of 0.84 mg/mL and 0.90 mg/mL, respectively, when compared to other combination groups. Unexpectedly, the FBN combination group (including anti-*FlaA* IgY, anti-*BabA2* IgY, and anti-*NapA* IgY) demonstrated relatively poor antibacterial effects. Therefore, these two combinations, including FUN and FUB, were then applied to subsequent antimicrobial experiments.

### 3.4 Inhibitory effect of IgY antibodies on *H. pylori* infection in GES-1 cells

**Figure 3:**
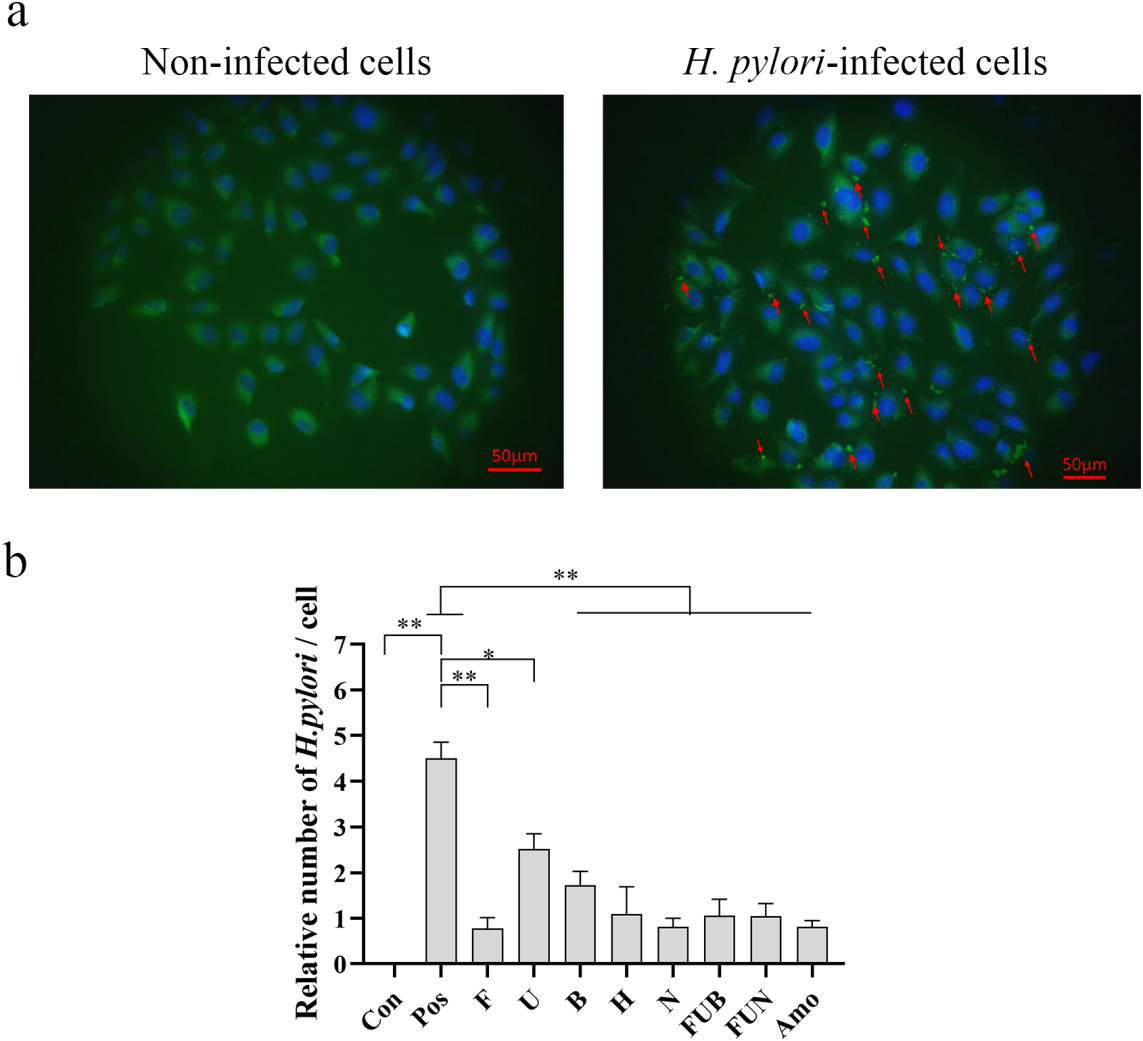
Inhibitory effects of IgY antibodies on *H*. *pylori* infection in GES-1 cells determined by immunofluorescence staining. After treatment with IgY antibodies, the *H*. *pylori* infection in GES-1 cells was evaluated using immunofluorescence staining. **(a)**, Representative images of the immunofluorescence staining under a fluorescence microscope (Scale bars, 50 μm). In the absence of *H*. *pylori* adhesion to GES-1 cells, no significant fluorescence signal is observed on the cell surface (left). In contrast, when bacteria adhere to the cells, a distinct fluorescent signal localizes to the cell surface (red arrow, right). **(b)** Quantitative analysis of *H*. *pylori* infection in GES-1 cells. The arrow indicates the adhesion of *H*. *pylori* to GES-1 cells. Con, non-infected cells; pos, *H*. *pylori-*infected cells; *F*, anti-*FlaA* IgY treatment; *U*, anti-*UreB* IgY treatment; *B*, anti-*BabA2* IgY treatment; *H*, anti-*HpaA* IgY treatment; *N*, anti-*NapA* IgY treatment; *FUB*, treatment with anti-*FlaA* IgY, anti-*UreB* IgY and anti-*BabA2* IgY combination; *FUN*, treatment with anti-*FlaA* IgY, anti-*UreB* IgY and anti-*NapA* IgY combination; Amo, amoxicillin treatment. Data are presented as mean ± SEM (n=4). *P < 0.05. **P < 0.01.

To investigate the preventive effect of IgY antibodies on *H. pylori* infection, the IgY antibodies were added to the well for co-incubation with GES-1 cells. As shown in **Figure 3**, *H. pylori* significantly adhered to and infected GES-1 cells. Each IgY antibody treatment could significantly inhibit the adhesion of *H. pylori* to the cells at various levels (p < 0.05). Among the single treatment groups, anti-*FlaA* IgY exhibited a better inhibitory effect on *H. pylori* infection, while anti-*UreB* IgY showed a moderate preventive effect. Additionally, the combination treatment groups, FUN and FUB, also exhibited similar inhibitory effects as anti-*FlaA* IgY on *H. pylori* infection at the same concentration (total 2.0 mg/mL).

### 3.5 Eradication rate of IgY treatment

**Figure 4:**
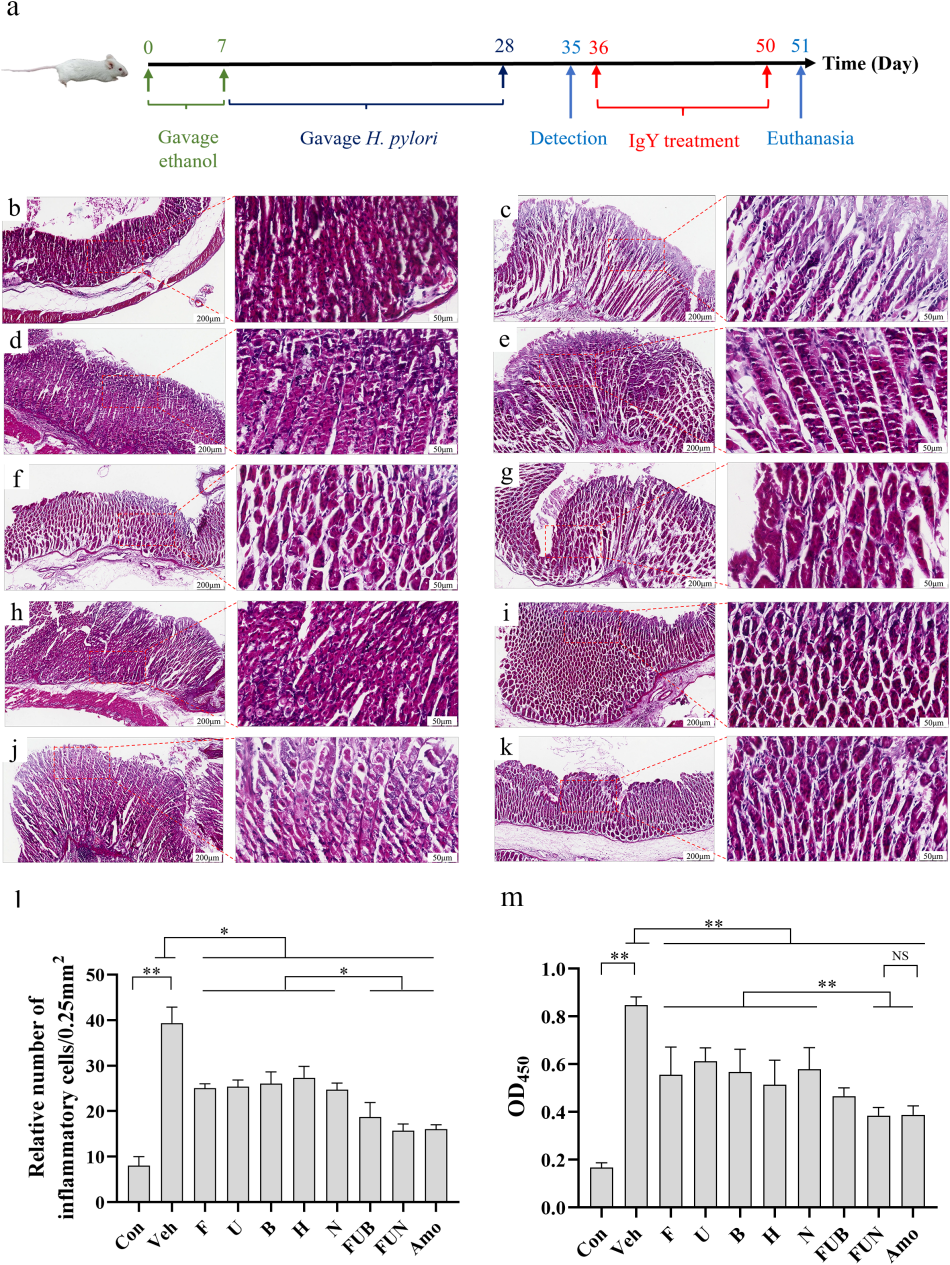
*H*. *pylori*-infected mice model establishment and IgY antibodies treatment. **(a)** Schematic diagram illustrating the establishment of the *H*. *pylori*-infected mice model and the treatment with IgY antibodies. Histopathological analysis of the gastric mucosa of mice infected with *H*. *pylori* (ATCC 700392), followed by treatment with IgY antibodies (20 mg·kg^−1^·day^−1^). Representative images of the gastric mucosa with H&E staining are displayed (Scale bars, 200 μm or 50 μm, as indicated in figures). Groups include: Normal mice group (Con) **(b)**, *H*. *pylori* + PBS vehicle group (Veh) **(c)**, *H*. *pylori* + anti-*FlaA* IgY group (F) **(d)**, *H*. *pylori* + anti-*UreB* IgY group (U) **(e)**, *H*. *pylori* + anti-*BabA2* IgY group (B) **(f)**, *H*. *pylori* + anti-*HpaA* IgY group (H) **(g)**, *H*. *pylori* + anti-*NapA* IgY group (N) **(h)**, *H*. *pylori* + FUB group (FUB) **(i)**, *H*. *pylori* + FUN group (FUN) **(j)**, and *H*. *pylori* + Amoxicillin group **(k)**. In comparison to the IgY-treated groups, the *H*. *pylori* + vehicle group exhibited altered gastric mucosal morphology, including degeneration of villi and infiltration of inflammatory cells. **(l)** Quantitative analysis for submucosal inflammatory cell infiltration. (n = 8). **(m)** Serum anti-*H. pylori* antibody detection by ELISA. (n = 8). *P < 0.05. **P < 0.01.

The strategy for *H. pylori* infection and IgY antibodies treatment in this study is shown in **Figure 4a**. After 3 weeks of *H. pylori* infection, the feces of the mice were tested using the rapid urease test (RUT), and the results indicated that 73.6% of the mice tested positive. Furthermore, 5 mice with a positive RUT test were randomly selected for *H. pylori* culture. The colonies on the plates were transparent, moist, and exhibited a flat morphology, staining red with Gram stain. These suspected colonies were confirmed as *H. pylori* through colony PCR (**Supplementary Figure 3**), indicating successful colonization of the gastric mucosa in the mice. In addition, compared with the normal control group, the *H. pylori*-infected group exhibited some characteristics of malaise, reduced appetite, constipation, and weight loss (p< 0.05) (**Supplementary Table 1**).

IgY treatment in each group achieved initial *H. pylori* eradication. Notably, anti-*FlaA* IgY, anti-*BabA2* IgY, and anti-*HpaA* IgY were the first confirmed to eradicate *H. pylori* infection, with eradication rates of 37.5%, 25%, and 25%, respectively. In addition, the eradication rate of *H. pylori* in both the FUB combination and the FUN combination was 37.5% (**Table 2**), while the eradication rate in the amoxicillin treatment group was 50%. Interestingly, after two weeks of IgY antibodies treatment, the diet and body weight of the mice significantly recovered compared to the vehicle (p < 0.05) (**Supplementary Table 1**).

**Table 2:** Eradication rate after dietary IgY antibodies in *H. pylori*-infected mice.

Group	Before treatment *H. pylori* ^+^	After treatment *H. pylori* ^+^	*H. pylori* eradication rate (%)
Normal	0	0	**/**
Vehicle	8	8	0.00
anti-*FlaA* IgY (F)	8	5	37.50
anti-*UreB* IgY (U)	8	6	25.00
anti-*BabA2* IgY (B)	8	6	25.00
anti-*HpaA* IgY (H)	8	6	25.00
anti-*NapA* IgY (N)	8	6	25.00
FUB	8	5	37.50
FUN	8	5	37.50
Amoxicillin	8	4	50.00

FUB, treatment with anti-*FlaA* IgY, anti-*UreB* IgY, and anti-*BabA2* IgY combination, with a total concentration of 20.0 mg·kg^−1^·day^−1^. FUN, treatment with anti-*FlaA* IgY, anti-*UreB* IgY, and anti-*NapA* IgY combination, with a total concentration of 20.0 mg·kg^−1^·day^−1^.

### 3.6 Histological findings

The gastric mucosal cells in non-infected mice were usually tightly packed, with intact tissue structure and clear lamina propria. The ratio of chief cells to parietal cells was within the normal range, and the morphology of these cells appeared intact, with inflammatory cell infiltrates being absent or rare. However, *H. pylori* infection caused significant damage to the gastric mucosa, degeneration of cell morphology, and pronounced inflammatory cell infiltration was observed. In single IgY treatment groups, the morphology of cells was comparatively intact, and the inflammatory cell infiltration and tissue damage were significantly reduced compared to the *H. pylori*-infected group. Interestingly, the FUN combination treatment significantly improved tissue repair and decreased immune cell infiltration, demonstrating effects similar to those of amoxicillin treatment (**Figures 4b-l**). In addition, IgY treatments—especially the FUN combination—exhibited a similar trend in significantly decreasing the antibody titer of anti-*H. pylori* serum compared to amoxicillin, suggesting that the *H. pylori* infection was significantly reduced after IgY treatments (**Figure 4m**).

### 3.7 Effects on gastric emptying rate and intestinal propulsion rate

**Figure 5:**
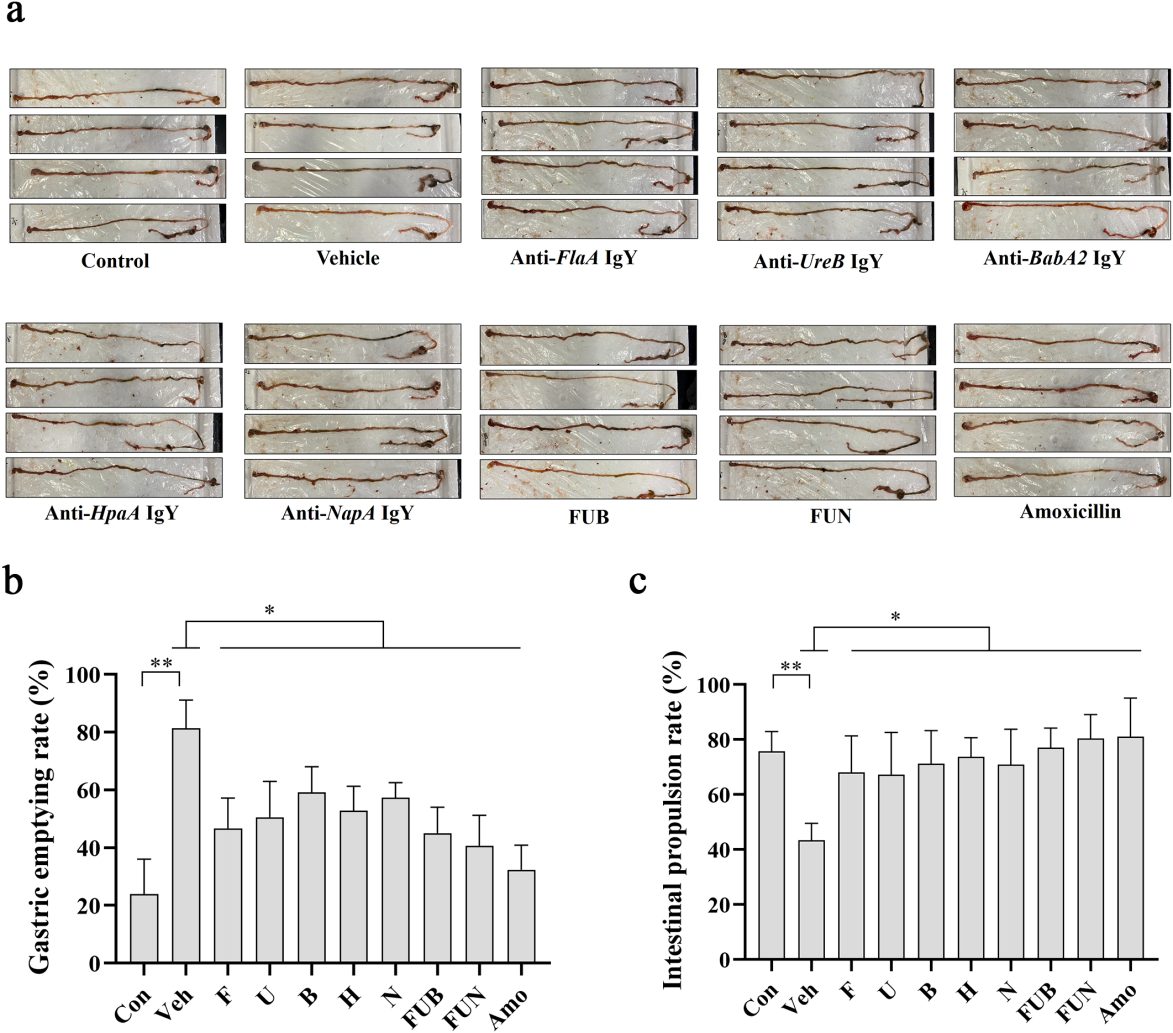
Gastric residual rate and intestinal propulsion rate. **(a)** Schematic diagram of the mouse intestinal tract after IgY treatments as indicated. The gastric residual rate **(b)** and intestinal propulsion rate **(c)** were calculated as described in the Materials and Methods section for each mouse in 10 groups, including normal mice group (Con), *H. pylori* + PBS vehicle group (veh), *H. pylori *+ anti-*FlaA* IgY group (F), *H. pylori* + anti-*UreB* IgY group (U), *H. pylori* + anti-*BabA2* IgY group (B), *H. pylori* + anti-*HpaA* IgY group (H), *H. pylori* + anti-*NapA* IgY group (N), *H. pylori*+ FUB group (FUB), *H. pylori* + FUN group (FUN), and *H. pylori* + Amoxicillin group (Amo). Data are presented as mean ± SEM (n = 8). * P < 0.05. ** P < 0.01.

*H. pylori* infection significantly impaired gastrointestinal function in mice by inhibiting gastric emptying and intestinal propulsion (**Figures 5a–c**). However, treatment with IgY antibodies notably restored gastrointestinal function in *H. pylori*-infected mice at various levels, improving the intestinal propulsion rate and reducing the gastric residual rate (p < 0.05). Notably, the combinations of IgY antibodies, specifically FUN and FUB, enhanced gastric emptying capacity and restored intestinal propulsion to levels comparable to normal mice (**Figures 5a–c**).

## 4 Discussion

In this study, we evaluated the antimicrobial roles of five IgY antibodies *in vivo* and *in vitro* within the same experimental system, confirming *FlaA* as a promising target against *H. pylori*. Although five IgY antibodies, including anti-*FlaA* IgY, anti-*BabA2* IgY, anti-*NapA* IgY, anti-*HpaA* IgY, and anti-*UreB* IgY, exhibited a directly inhibitory role on *H. pylori* growth and adhesion, their antimicrobial effects varied *in vitro*. The direct effects of these IgY antibodies on *H. pylori* growth were ranked as follows: anti-*FlaA* IgY > anti-*BabA2* IgY > anti-*NapA* IgY > anti-*HpaA* IgY ≈ anti-*UreB* IgY, while the effects on *H. pylori* infection to GES-1 cells were ranked as anti-*FlaA* IgY ≈ anti-*NapA* IgY > anti-*HpaA* IgY > anti-*BabA2* IgY > anti-*UreB* IgY. Among these IgY antibodies, the anti-*FlaA* IgY exhibited an obvious advantage over other individual antibodies against *H. pylori* infection.

Additionally, we identified two effective combinations of IgY antibodies: FUN and FUB. These combinations showed significant advantages in inhibiting the growth and infection of *H. pylori* while promoting the recovery of gastrointestinal function at the same dosage. Although IgY antibodies have exhibited significant efficacy in recent clinical studies targeting *H. pylori* infection (17–19), their therapeutic effectiveness remains inferior to that of traditional antibiotic treatments. Therefore, based on the different functions and roles of genes during *H. pylori* infection, employing a combination strategy with IgY antibodies to enhance their anti-*H. pylori* efficacy is a crucial focus in developing IgY antibody-based drugs. *BabA2*, *HpaA*, and *NapA* are important adhesins of *H. pylori* that promote the infection of cells by *H. pylori*. *HpaA* is expressed in almost all *H. pylori* (20), and the anti-*HpaA* IgY exhibits antibacterial adhesion effects similar to those of anti-*BabA2* IgY in our test. *BabA2* is a crucial adhesin that shows a high rate of positive serum antibody detection in patients with *H. pylori* infection (21). Antibodies or vaccines targeting *BabA2* can effectively block *H. pylori* infection and significantly reduce the incidence of ulcers and gastric cancer (22). Therefore, *BabA2* was included in the antibody combination for our animal experiments. *UreB* is widely expressed in *H. pylori* and is considered one of the first targets for developing IgY antibodies. Its effectiveness has been validated in both animal and human clinical trials (13, 23). In this study, anti-*UreB* IgY exhibited relatively low activity against *H. pylori* growth and adhesion *in vitro* compared to other antibodies. However, it demonstrated similar *H. pylori* eradication effects to the other IgY antibodies in *in vivo* assays, which may be attributed to its gastric acid-neutralizing effect (24). Therefore, *UreB* was included in the antibody combination for *in vivo* experiments. Based on these considerations, we designed and identified two combinations with enhanced anti-*H. pylori* efficacy, namely FUN and FUB, which targeted different processes related to bacterial colonization, including blocking movement, creating a suitable environment, and inhibiting adhesion.

Notably, factors such as target selection, IgY antibody dosage, and treatment duration ​may be key factors affecting the antimicrobial activity of IgY in initial *Helicobacter pylori* eradication. Firstly, different genes play distinct roles in the infection and colonization processes of *H. pylori*. Therefore, it is hypothesized that IgY antibodies targeting different genes should exhibit varying antibacterial efficacies. Findings from this study revealed differences in the inhibitory effects of IgY antibodies targeting five different colonization-related genes, with anti-*FlaA* IgY demonstrating the strongest antibacterial activity. Urease was the earliest target developed for *H. pylori*-specific IgY. Both animal studies and human tests have shown that urease-targeted IgY significantly reduces *H. pylori* infection load, promotes gastric mucosal repair, and alleviates clinical symptoms (13, 14, 25). However, oral administration of urease-targeted egg yolk antibodies (IgY) alone fails to achieve clinical eradication of *H. pylori* (eradication rate: 0%) (25). Therefore, developing multivalent IgY that simultaneously targets multiple genes is necessary to achieve complementary or synergistic effects, thereby enhancing IgY’s efficacy in eradicating *H. pylori* infections. In this study, we identified two combinations, FUB and FUN, which not only demonstrated antimicrobial advantages but also promoted gastrointestinal recovery in mice, confirming the feasibility of combination therapy against *H. pylori* infection.

In addition to gene targets, the dosage of IgY significantly impacts its antibacterial efficacy (26). In this study, the antimicrobial activity of single IgY treatment exhibited a significant dose-dependent characteristic (**Figure 2**). In the previous study, a 30.8% first eradication rate was achieved with the oral administration of 100 mg of IgY once daily (16), while Hao et al. reported an eradication rate of 50.74% with a dosage of 250 mg of IgY taken orally twice daily (18). Although there are some differences in the targets, high doses are beneficial for the eradication of *H. pylori in vivo*. Notably, the total dosage of the antibody combination was designed to be consistent with that of single antibody treatments in our tests. However, the antibacterial effects and promotion of gastrointestinal function recovery were superior to those of single antibody treatments, potentially indicating a synergistic effect between IgY antibodies. Therefore, future studies should fully consider the characteristics of the targets and the dosage of IgY antibodies to achieve the best eradication effect against *H. pylori*.

Finally, the duration of oral IgY administration represents another critical yet underexplored variable. Unfortunately, no clinical evidence currently exists regarding the impact of prolonged IgY treatment on *H. pylori* eradication efficacy. Our group has initiated systematic investigations to address this question. In addition, there are some limitations in this study, including the prepared IgY not representing the effective concentration of respective antigen-specific antibodies due to the lack of affinity purification. To address this limitation and ensure the accuracy of our results, we implemented the following measures: first, we standardized the antibody preparation protocol, including consistent antigen immunization dosage, administration method, and extraction procedures; second, we included a PBS-immunized control group, in which PBS-IgY antibody was prepared identically and used as a negative control in antibody titer assays (**Figure 1c**) and *in vitro* antibacterial evaluations (**Figures 2a, b**).

## 5 Conclusion

These IgY antibodies against colonization-related genes show a direct role in blocking the growth and infection of *H. pylori*, and their combination treatment could offer greater benefits against *H. pylori* infection and promote the recovery of gastrointestinal function.

## Data Availability

The original contributions presented in the study are included in the article/**Supplementary Material**, further inquiries can be directed to the corresponding author/s.
