# Supplementary material for: Immunoglobulin Y antibodies against colonization-related genes block the growth and infection of Helicobacter pylori
Source: Front Immunol. 2025 Jun 18;16:1582250. doi: 10.3389/fimmu.2025.1582250 (PMC12213714; doi:10.3389/fimmu.2025.1582250)
Supplement: Supplementary file 1 [file Table1.docx]

Supplementary Material

# Supplementary Data

1. *FlaA* (GenBank: AY714225.1):

ATGGCTTTTCAGGTCAATACAAATATCAATGCGATGAATGCGCATGTGCAATCCGCACTCACTCAAAATGCGCTTAAAACTTCATTGGAGAGATTGAGTTCAGGTTTAAGGATTAATAAAGCGGCTGATGATGCATCAGGCATGACGGTGGCAGATTCTTTGCGTTCACAAGCGAGCAGTTTGGGTCAAGCGATTGCCAACACGAATGACGGCATGGGGATTATCCAAGTTGCGGATAAGGCTATGGATGAGCAGTTAAAAATCTTAGACACCGTTAAGGTTAAAGCGACTCAAGCGGCTCAAGACGGGCAAACTACGGAATCTCGTAAAGCGATTCAATCTGACATCGTTCGTTTGATTCAAGGTTTAGATAATATCGGTAACACGACTACTTATAACGGGCAAGCGTTATTGTCTGGTCAATTCACTAACAAAGAATTCCAAGTAGGGGCTTATTCTAACCAAAGCATTAAGGCTTCTATCGGCTCTACCACTTCGGATAAAATCGGTCAGGTTCGTATCGCTACAGGCGCGTTAATCACGGCTTCTGGGGATATTAGCTTGACTTTTAAACAAGTGGATGGCGTGAATGATGTAACTTTAGAGAGCGTAAAAGTTTCTAGTTCAGCAGGCACGGGGATCGGTGTGTTAGCGGAAGTGATTAACAAAAATTCTAACCGAACAGGGGTTAAAGCTTATGCGAGCGTTATCACCACGAGCGATGTGGCGGTCCAATCAGGAAGTTTGAGTAATTTAACTTTAAATGGGATCCATTTGGGTAATATCGCAGATATTAAGAAAAATGACTCAGACGGAAGGTTAGTCGCAGCGATCAATGCGGTTACTTCAGAAACCGGCGTGGAAGCTTATACGGATCAAAAAGGGCGCTTGAATTTGCGCAGTATAGATGGTCGTGGGATTGAAATCAAAACCGATAGCGTCAGTAATGGGCCTAGTGCTTTAACGATGGTCAATGGCGGTCAGGATTTAACAAAAGGTTCTACTAACTATGGGAGGCTTTCTCTCACACGCTTAGACGCTAAAAGCATCAATGTCGTTTCGGCTTCTGATTCGCAACATTTAGGTTTCACAGCGATTGGTTTTGGGGAATCTCAAGTGGCAGAAACCACGGTGAATTTGCGCGATGTTACTGGGAATTTTAACGCTAATGTCAAATCAGCCAGTGGCGCGAACTATAACGCCGTGATCGCTAGCGGTAACCAAAGCTTGGGATCTGGGGTTACAACCTTGAGAGGCGCGATGGTGGTGATTGATATTGCGGAATCGGCGATGAAAATGTTGGATAAAGTCCGCTCTGATTTAGGTTCTGTGCAAAATCAAATGATTAGCACCGTGAATAACATCAGCATCACTCAAGTGAATGTTAAAGCGGCTGAGTCTCAAATCAGGGATGTGGATTTTGCTGAAGAGAGCGCGAATTTCAATAAAAACAATATTTTGGTGCAATCAGGCAGCTATGCGATGAGTCAAGCTAACACCGTCCAACAAAATATCTTAAGGCTTTTAACTTAG

1. *NapA* (GenBank: Y714227.1):

ATGAAAACATTTGAAATTTTAAAACATTTGCAAGCGGATGCGATCGTGTTATTTATGAAAGTGCATAACTTCCATTGGAATGTGAAAGGCACCGATTTTTTCCATGTGCATAAAGCCACTGAAGAAATTTATGAAGAATTTGCGGACATGTTTGATGATCTCGCTGAAAGGATTGTTCAATTAGGACACCACCCATTAGTCACTTTATCCGAAGCGCTCAAACTCACTCGCGTCAAAGAAGAAACTAAAACAAGCTTCCACTCTAAAGACATCTTTAAAGAAATTCTAGGCGATTACAAACACCTAGAAAAAGAATTTAAAGAGCTCTCTAACACCGCTGAAAAAGAAGGCGATAAAGTAACCGTAACTTATGCGGACGATCAATTAGCCAAGTTGCAAAAATCCATTTGGATGCTAGAAGCCCATTTGGCTTAA

1. *HpaA* (GenBank: Y714223.1):

ATGAGAGCAAATAATCATTTTAAAGATTTTGCATGGAAAAAATGCCTTTTAGGCGCGAGCGTGGTGGCTTTGTTGGTGGGATGCAGCCCGCATATTATTGAAACCAATGAAGTCGCTTTGAAATTGAATTACCATCCAGCTAGCGAGAAAGTTCAAGCGTTAGATGAAAAGATCTTGCTTTTAAGGCCAGCTTTTCAATACAGCGATAATATTGCTAAAGAGTATGAAAACAAATTCAAGAATCAAACCACGCTTAAGGTTGAAGAGATCTTGCAAAATCAAGGCTATAAGGTTATTAGCGTAGATAGCAGCGATAAAGACGATCTTTCTTTTTCGCAAAAAAAAGAAGGGTATTTGGCCGTCGCTATGAATGGCGAAATTGTTTTACGCCCCGATCCTAAAAGGACCATACAGAAAAAATCAGAACCCGGGTTATTATTCTCCACTGGTTTGGACAAAATGGAAGGGGTTTTAATCCCGGCTGGGTTTGTCAAGGTTACCATACTAGAGCCTATGAGTGGGGAATCTTTAGATTCTTTTACGATGGATTTGAGCGAGTTGGACATTCAAGAAAAATTCTTAAAAACCACCCATTCAAGCCATAGCGGGGGGTTAGTTAGCACTATGGTTAAGGGAACGGATAATTCTAATGACGCGATCAAGAGCGCTTTGAATAAGATTTTTGCAAATATCATGCAAGAAATAGACAAAAAGCTCACTCAAAAGAATTTAGAATCTTATCAAAAAGACGCCAAGGAATTGAAAAACAAGAGAAACCGATAA

1. *UreB* (GenBank: CP071982.1):

ATGAAAAAGATTAGCAGAAAAGAATATGTTTCTATGTATGGCCCTACCACAGGCGATAAAGTGAGATTGGGCGATACAGATTTGATTGCTGAAGTAGAACATGACTACACCATTTATGGCGAAGAGCTTAAATTCGGTGGCGGTAAAACCCTAAGAGAAGGCATGAGCCAATCCAACAACCCTAGCAAAGAAGAACTGGATTTAATCATCACTAACGCTTTAATCGTGGATTACACCGGTATTTATAAAGCGGATATTGGTATTAAAGACGGCAAAATCGCTGGCATTGGTAAAGGCGGTAACAAAGACATGCAAGATGGCGTTAAAAACAATCTTAGCGTGGGTCCTGCTACTGAAGCACTAGCCGGTGAAGGCTTGATCGTAACGGCTGGTGGTATTGACACACACATCCACTTCATTTCACCCCAACAAATCCCTACAGCTTTTGCAAGCGGTGTAACAACCATGATTGGTGGCGGAACCGGTCCTGCTGATGGCACTAATGCGACTACTATCACTCCAGGTAGAAGAAACTTAAAATGGATGCTCAGAGCAGCTGAAGAATATTCTATGAACTTAGGTTTCTTAGCTAAAGGTAACACTTCTAACGACGCGAGCTTAGCGGATCAAATTGAAGCCGGTGCGATTGGCTTTAAAATCCACGAAGACTGGGGAACCACTCCTTCTGCAATCAATCATGCGTTAGATGTTGCAGACAAATACGATGTGCAAGTCGCTATCCACACAGACACTTTGAATGAAGCCGGTTGCGTGGAAGACACTATGGCAGCTATTGCCGGACGCACTATGCACACTTTCCACACTGAAGGCGCTGGCGGCGGACACGCTCCTGATATTATTAAAGTGGCCGGTGAACACAACATCCTACCCGCTTCCACTAACCCCACTATCCCTTTCACCGTGAATACAGAAGCCGAACACATGGACATGCTTATGGTGTGCCACCACTTGGATAAAAGCATTAAAGAAGATGTCCAGTTCGCTGATTCAAGGATCCGTCCTCAAACCATTGCGGCTGAAGACACTTTGCATGACATGGGGATTTTCTCCATCACCAGTTCTGACTCTCAAGCGATGGGTCGTGTGGGTGAAGTCATCACTAGAACTTGGCAAACAGCTGACAAAAACAAAAAAGAATTTGGCCGCTTGAAAGAAGAAAAAGGCGATAACGACAATTTCAGGATCAAACGCTACTTGTCTAAATACACCATTAACCCAGCGATCGCTCATGGGATTAGCGAGTATGTAGGTTCAGTAGAAGTGGGCAAAGTGGCTGACTTGGTATTGTGGAGTCCAGCATTCTTTGGCGTGAAACCCAACATGATCATCAAAGGCGGATTCATTGCATTAAGCCAAATGGGCGATGCGAACGCTTCTATCCCTACCCCACAACCGGTTTATTACAGAGAAATGTTCGCTCACCATGGTAAAGCTAAATACGATGCAAACATCACTTTTGTGTCTCAAGCGGCTTATGACAAAGGCATTAAAGAAGAATTAGGGCTTGAAAGACAAGTGTTGCCGGTTAAAAATTGCAGAAACATCACTAAAAAAGACATGCAATTCAACGACACTACCGCTCACATTGAAGTCAATCCTGAAACTTACCATGTGTTCGTGGATGGCAAAGAAGTCACTTCTAAACCAGCCAATAAAGTGAGCTTGGCTCAACTCTTTAGCATTTTCTAG

1. *BabA2* (GenBank: 071982.1):

GCTGAAGACGACGGCTTTTACACAAGCGTAGGCTATCAAATCGGTGAAGCCGCTCAAATGGTATCAAACACCAAAGGCATCCAACAGCTTTCAGACAACTATGAAAACTTGAGCAAGCTTTTAACCAGATACAGCACCCTAAACAACTTCATCCAATTGGCCTCTGACCCGAGTGCGATCAACGCGGCTCGTGAAAATCTGGGTGCGAGCGCGAAGAACTTGATTGGCGATACCAAAAATTCCCCAGCCTATCAAGCCGTGCTTTTAGCGATCAATGCGGCGGTAGGGTTTTGGAATGTCGTAGGCTATGTTACGCAATGCGGGGGTAATGGTAATGAAAAAAGCACCTCTTCAACCACCATCTTCAACAACGAGCCAGGGTATCGATCCACTTCCATCACTTGTTCTTTGAACAGATATTCTCCTGGATACTATGGCCCTATGGGTATTGATAATTTTAAAAAGCTTAACGAAGCCTATCAAATCCTCCAAGCGGCTTTAAAGAAAGGCTTACCCGCACTCAAAAACAACAACGGGACGCTCGGAGAAGTAAAATATACTTACACATGCTCAGGGGAAGGGAATACTAACTGCAATGCGCTAGTGGGCAATGGAAACGGAGAAGATAGACGAAACGGCGGAACCAAGACTGAAATCCAAACCATAGACGGCAAAACCGTAACCACCACGATCAGCTCAAAAGTGGTTAATTATGGAGCGGCAGGTAACACTCAAGGTGTGTCCTACACCGAAATCACTAACGCATTGAAAGGTGTGCCTGATAGCGCTCAAGCGCTCTTAGCGCAAGCGAGCACGCTCATCAACACCATCAACGAAGCATGCCCGTTTTTCCATGCGCCTAATAGCCAAGCTGGTGGCCCAAAATGGGAATGGCCCTCTAACAAGCTGTGCGGCGCTTTTTCACAAGAAATCAGCGCGATCCAAAAGATGATCACAGACGCGCAAGAGCTTGTCAATCAAACGAGTATCATTAATAGTGATGAGCAAACCGCTCAAGTGGGAGGCAGTGGAGGCAAGCCTTTCAACCCTTTCACAGACGCTAGCTTTGCGCAAGGCATGCTCAAAAACGCTAGCGCGCAAGCCAAAATGCTCGATTTAGCCCATCAAGTGGGGCAAACCATTAACCCTAGCAATCTTAGCGGGACTTTTAAAAATTTTGTTACAGGCTTTTTAGCCACATGCAACAACCCCTCAACAGCTGGCACTGGTAGCACACAAGGTTCGGCTCCTGGCACAGTGACCACTCAA

# Supplementary Figures and Tables

**Supplementary Table 1 The changes in body weight before and after dietary IgY antibodies**

| Group | Before *H. pylori* infection (14d) | Before IgY  treatment (35d) | After IgY treatment  (56d) |
| --- | --- | --- | --- |
| Normal | 25.94±1.64 | 29.34±0.89 | 31.46±0.95**^b^** |
| Vehicle | 26.47±1.65 | 25.81±1.45**^a^** | 25.54±1.83**^a^** |
| anti-*FlaA* IgY | 26.58±1.34 | 25.64±1.61**^a^** | 27.68±1.79**^a^** |
| anti-*UreB* IgY | 25.23±1.26 | 24.63±0.64 | 26.84±1.47**^a^** |
| anti-*BabA2* IgY | 26.81±0.90 | 25.11±0.80**^a^** | 28.11±1.56**^a^** |
| anti-*HpaA* IgY | 26.15±0.95 | 24.98±1.52**^a^** | 27.96±1.92**^a^** |
| anti-*NapA* IgY | 26.20±1.05 | 25.38±1.78**^a^** | 27.01±3.84**^a^** |
| FUB | 26.34±0.84 | 24.99±1.97**^a^** | 28.49±1.56**^ab^** |
| FUN | 26.95±1.31 | 24.68±0.96 | 28.35±1.31^a^**^b^** |
| Amoxicillin | 27.05±0.96 | 24.43±1.04 | 29.74±1.41**^b^** |

Note: Data are presented as mean ± SEM (n=8). FUB, combination treatments with anti-*FlaA* IgY, anti-*UreB* IgY, and anti-*NapA* IgY in a ratio of 1:1:1, total 20.0 mg/(kg·day). FUN, combination treatments with anti-*FlaA* IgY, anti-*UreB* IgY, and anti-*BabA2* IgY in a ratio of 1:1:1, total 20.0 mg·kg⁻¹·day⁻¹. **^a^** P < 0.05 vs. “Normal”; **^b^** P < 0.05 vs. “Vehicle”.


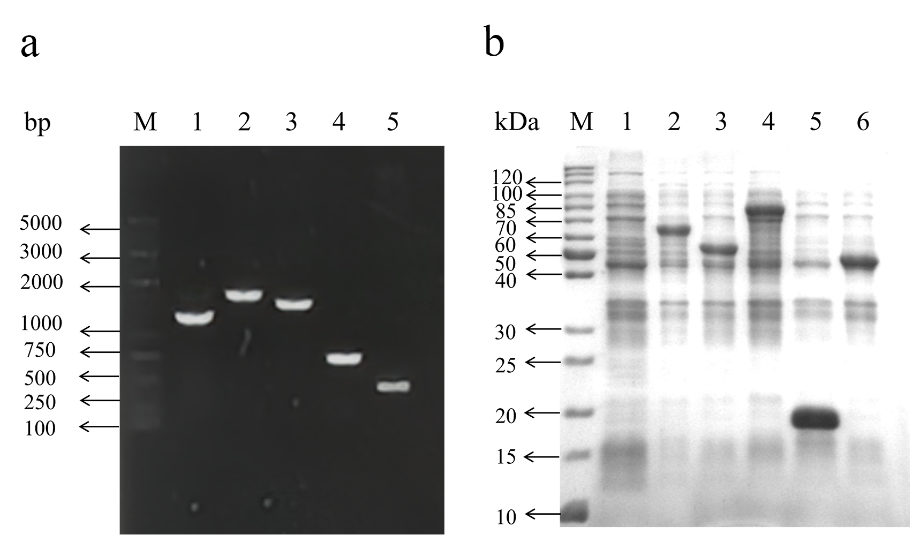


**Supplementary Figure 1. Preparation of recombinant antigens**. (a) Identification by Colony PCR. Lane M: DL5000 DNA Marker; Lanes 1 to 5: recombinant genes for *BabA2*, *UreB*, *FlaA*, *HpaA*, and *NapA*, respectively. (b) SDS-PAGE analysis of recombinant antigen expression. Lane M: standard protein marker; Lane 1: before induction with IPTG; Lanes 2 to 6: expressions of *BabA2*, *FlaA*, *UreB*, *NapA*, and *HpaA* under IPTG induction, respectively.


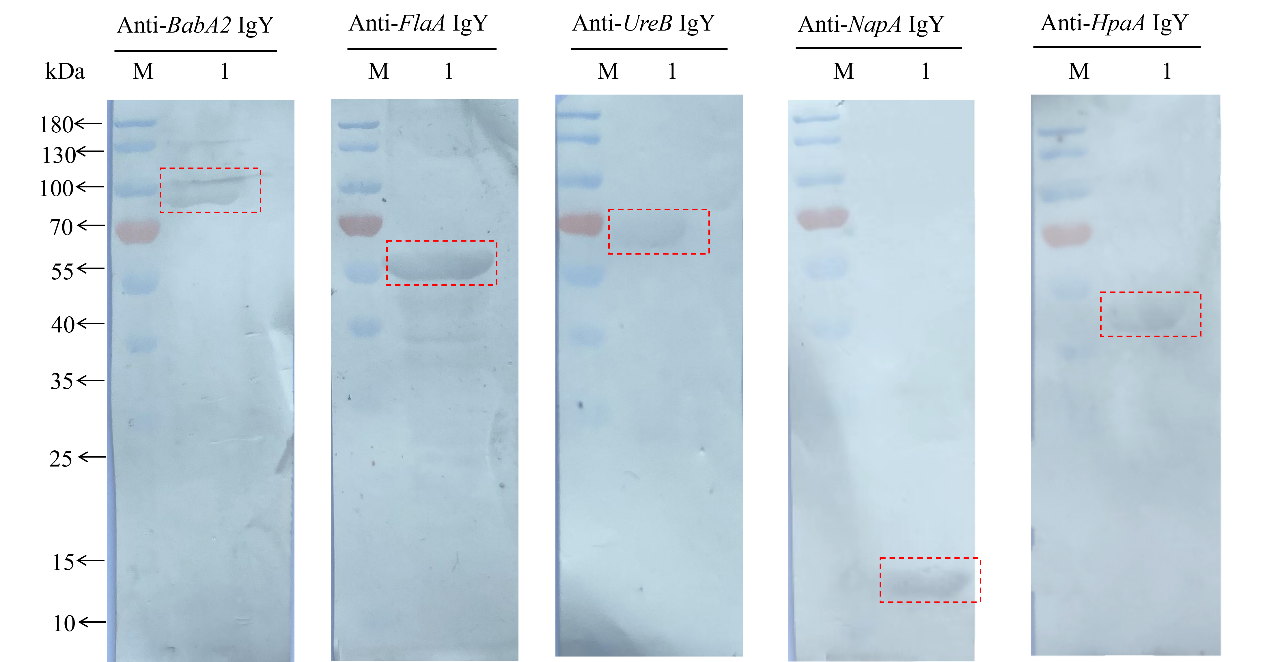


**Supplementary Figure 2.** **The results of western blotting with IgY antibodies**. The binding abilities of IgY antibodies, including anti-*BabA2* IgY, anti-*FlaA* IgY, anti-*UreB* IgY, anti-*NapA* IgY, and anti-*HpaA* IgY, to the recombinant antigens. Lane M is the standard protein marker; Lane 1 is the recombinant antigens.

**
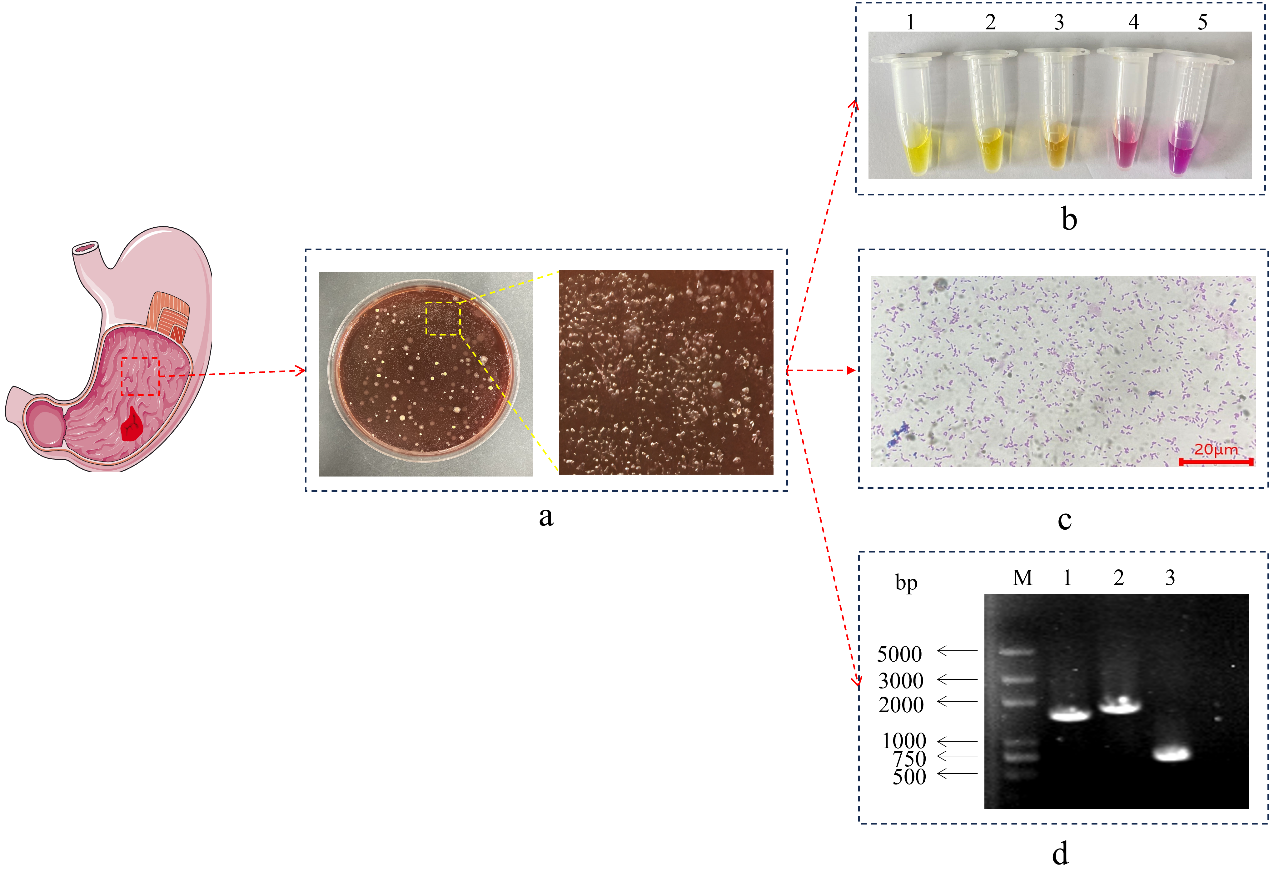
**

**Supplement Figure 3.** **Detection of the *H. pylori*-infected mice model.** (a) Results of culturing *H. pylori* using gastric mucosa from mice gavaged with *H. pylori*. (b) Rapid urease test (RUT) is used to determine the presence of *H. pylori* in cultured gastric mucosa. Tube Nos. 1 to 5: five suspected colonies of *H. pylori.* (c) Results of Gram staining of suspected *H. pylori* colonies. Scale Bar =20 μm.(d) Identification by colony PCR. Lane M: DNA marker; lanes 1 to 3: PCR products with primers targeting *UreB*, *FlaA*, and *HpaA*, respectively.

stomach-ulcer icon by Servier https://smart.servier.com/ is licensed under CC-BY 3.0 Unported <https://creativecommons.org/licenses/by/3.0/>
